# Sequential label shift detection in classification data: An application to dengue fever

**DOI:** 10.1371/journal.pone.0310194

**Published:** 2024-09-16

**Authors:** Ciaran Evans, Max G’Sell

**Affiliations:** 1 Department of Statistical Sciences, Wake Forest University, Winston-Salem, NC, United States of America; 2 Department of Statistics & Data Science, Carnegie Mellon University, Pittsburgh, PA, United States of America; Instituto Nacional de Salud Pública: Instituto Nacional de Salud Publica, MEXICO

## Abstract

Classifiers have been developed to help diagnose dengue fever in patients presenting with febrile symptoms. However, classifier predictions often rely on the assumption that new observations come from the same distribution as training data. If the population prevalence of dengue changes, as would happen with a dengue outbreak, it is important to raise an alarm as soon as possible, so that appropriate public health measures can be taken and also so that the classifier can be re-calibrated. In this paper, we consider the problem of detecting such a change in distribution in sequentially-observed, unlabeled classification data. We focus on label shift changes to the distribution, where the class priors shift but the class conditional distributions remain unchanged. We reduce this problem to the problem of detecting a change in the one-dimensional classifier scores, leading to simple nonparametric sequential changepoint detection procedures. Our procedures leverage classifier training data to estimate the detection statistic, and converge to their parametric counterparts in the size of the training data. In simulated outbreaks with real dengue data, we show that our method outperforms other detection procedures in this label shift setting.

## Introduction

Dengue fever is a viral infection which affects up to 400 million people a year [1]. To improve diagnosis, several authors have developed classifiers based on simple diagnostic and laboratory measurements, such as temperature, vomiting, and white blood cell count [2–4]. Such a classifier will necessarily be applied sequentially, making a prediction for each new patient with possible dengue symptoms, while the true dengue status may remain unobserved. However, the prevalence of dengue in a community may change quickly, due to both seasonal trends and outbreaks [5–7]. We need to detect this change as soon as possible, because a change in community prevalence impacts the quality of our classifier predictions, and also as a matter of public health.

In this paper, we propose a method to detect changes in sequentially-observed classification data, by directly using classifier predictions to construct a detection statistic. We apply our approach to simulated outbreaks of different speeds and severities, using existing dengue classification data from [3], and demonstrate competitive performance compared to other changepoint detection procedures.

In the case of a dengue outbreak, we have (a) a stream of unlabeled data (new patients) which require predictions, (b) a classifier making predictions sequentially on the new data, and (c) a change in the distribution of new data (i.e., the outbreak). To detect changes in the prevalence of dengue fever, our work leverages the *label shift* assumption, in which the marginal distribution of the labels changes, but the conditional distributions of the features (given the label) do not. In the dengue setting, label shift occurs if the prevalence of dengue changes but the symptoms of the disease do not, and label shift has previously been proposed as a reasonable mechanism for changes in disease prevalence [8]. From a public health perspective, an assumption like label shift is valuable because it allows us to characterize the type of change we expect to see in the population, and thereby develop a method to target this change.

Our work proposes a nonparametric procedure for detecting label shift by calculating a detection statistic with each new classifier prediction. That is, by using classifier predictions, we do not need to specify the underlying distribution of the observed data. Our method leverages the label shift assumption directly, and outperforms other nonparametric procedures when the label shift assumption is approximately correct, while requiring less knowledge about the data distributions than optimal procedures.

Below, we formally define the problem of detecting label shift in dengue data, and review existing changepoint detection methods. We then describe our proposed label shift detection procedure. Through simulations, we show that our proposed method outperforms other detection procedures when the label shift assumption holds, and can still detect changes even when the label shift assumption is violated; this is consistent with the work of [9], who showed that two-sample tests for label shift perform well for detecting other, more general changes in distribution. To demonstrate performance of our procedure with real classifier data, we use real dengue data from [3] and simulate a variety of changes in dengue prevalence. All code and data to reproduce the analysis in this paper is available at https://github.com/ciaran-evans/label-shift-detection.

## Methods

### Motivation: Dengue fever

Dengue, a viral infection transmitted by mosquitoes, is found in tropical and sub-tropical regions around the world, and affects up to 400 million people a year [1]. Diagnosis of dengue is important for the patient to receive appropriate treatment, and early treatment can improve prognosis. However, dengue cases are commonly mis-diagnosed [1]; while gold-standard diagnostic tests and rapid antigen tests exist, these may not always be available to healthcare providers. To assist healthcare workers in diagnosis and early detection of dengue [3], developed a classifier based on simple diagnostic and laboratory measurements, such as temperature, vomiting, and white blood cell count. The authors recommend deploying the classifier to help diagnose dengue in patients, which entails sequentially applying the classifier to make a prediction for each new patient.

However, the prevalence of dengue in a community may change quickly, due to both seasonal trends and outbreaks [5–7]. When a sudden change in dengue prevalence occurs, it is vital to raise an alarm; as noted by [7], “strategies are needed to respond quickly to unexpected incidents.”

We apply our changepoint detection procedure to the problem of detecting a change in the prevalence of dengue, using data and classifier predictions from the work of [3]. As the prevalence of dengue changes, but the symptoms are expected to stay the same, the label shift assumption is appropriate for this change. We have simulated the changes in dengue prevalence to explore a variety of different changes, but the data used for simulation are real, and the classifier is adapted from [3].

### Problem statement

To formally define the problem and our proposed method, some notation is needed. Suppose (*X*_1_, *Y*_1_), (*X*_2_, *Y*_2_), $\left(X_{3},Y_{3}\right),\ldots \in R^{d}\times \left\{0,1\right\}$ is a sequence of feature vectors *X*_*i*_ and associated *unobserved* binary labels *Y*_*i*_. In our dengue example, *X*_*i*_ represents diagnostics measurements like white blood cell count and platelet count, while *Y*_*i*_ represents true dengue status. We emphasize here that while the feature vectors *X*_*i*_ are observed, the true labels *Y*_*i*_ are unobserved. That is, if we are diagnosing dengue fever, we observe the symptoms but not the true disease status.

**Definition 1** (Changepoint). Suppose that at some time *ν* ≥ 0, the joint distribution of (*X*_*i*_, *Y*_*i*_) changes, with $\left(X_{1},Y_{1}\right),\ldots ,\left(X_{\nu },Y_{\nu }\right)\overset{iid}{∼}P_{\infty }$ before the change, and $\left(X_{\nu +1},Y_{\nu +1}\right),\left(X_{\nu +2},Y_{\nu +2}\right),\ldots \overset{iid}{∼}P_{0}\neq P_{\infty }$ after the change occurs. (For example, a dengue outbreak causes an increase in the rate of positive cases). We call *ν* the *changepoint*.

Our aim is to detect this change in the distribution of (*X*_*i*_, *Y*_*i*_), using only the observed *X*_*i*_; the general problem of detecting that a change has occurred in sequentially-observed data is called *sequential changepoint detection*, and we discuss mathematical details below. In this case, we are aided by a labeled training set $\left(X_{1}^{\prime },Y_{1}^{\prime }\right),\ldots ,\left(X_{m}^{\prime },Y_{m}^{\prime }\right)\overset{iid}{∼}P_{\infty }$. The assumption that the observations are independent is common in many changepoint detection methods, and is used when characterizing the performance of the detection procedure.

Because arbitrary changes to high-dimensional classification data (that is, data with many features recorded for each observations) may be impossible to correct or detect, it is standard to make additional assumptions on the nature of the change. Because it frequently arises in practice, we will focus on the **label shift** setting [10, 11], which has received recent attention in the machine learning literature [8, 9, 12, 13]. Label shift assumes that the marginal distribution of *Y* changes, but the conditional distribution of *X*|*Y* does not:

**Definition 2** (Label shift). Let *f*_∞,*X*,*Y*_, *f*_∞,*Y*_, *f*_∞,*X*_, and *f*_∞,*X*|*Y*=*y*_ denote the probability functions of (*X*, *Y*), *Y*, *X*, and *X*|*Y* = *y* respectively, under $P_{\infty }$. Similarly define *f*_0,*X*,*Y*_, *f*_0,*Y*_, *f*_0,*X*_, and *f*_0,*X*|*Y*=*y*_. The label shift assumption is that *f*_0,*X*|*Y*=*y*_ ≡ *f*_∞,*X*|*Y*=*y*_ for all *y*, so

$$\begin{array}{c} f_{0,X,Y}\left(x,y\right)=f_{0,Y}\left(y\right)f_{0,X|Y=y}\left(x\right)=f_{0,Y}\left(y\right)f_{\infty ,X|Y=y}\left(x\right)\;\forall x,y, \end{array}$$ (1)

and

$$\begin{array}{c} \begin{array}{cc} f_{\infty ,X}\left(x\right) & =\sum_{y}f_{\infty ,Y}\left(y\right)f_{\infty ,X|Y=y}\left(x\right)\\ f_{0,X}\left(x\right) & =\sum_{y}f_{0,Y}\left(y\right)f_{0,X|Y=y}\left(x\right)=\sum_{y}f_{0,Y}\left(y\right)f_{\infty ,X|Y=y}\left(x\right). \end{array} \end{array}$$ (2)

**Fig 1:**
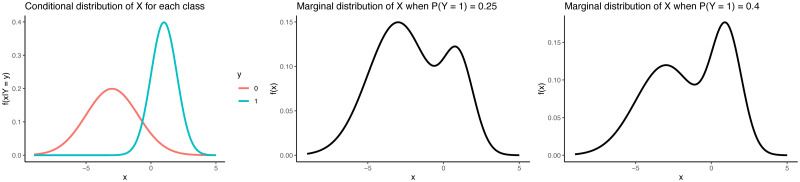
Univariate example of label shift. The left-hand panel shows the conditional distributions *f*_∞,*X*|*Y*=*y*_(*x*) for *y* = 0 and *y* = 1; these conditional distributions are the same for pre- and post-change data. The middle panel shows the marginal distribution *f*_∞,*X*_ when *f*_∞,*Y*_(1) = 0.25, and the right-hand panel shows the marginal distribution *f*_0,*X*_ when *f*_0,*Y*_(1) = 0.4.

Label shift is simply a change in the mixing proportion for the class distributions *X*|*Y* = 0 and *X*|*Y* = 1. In the dengue example, label shift implies that the symptoms *X* have a common distribution conditional on the dengue status *Y*, while the prevalence of dengue cases changes. An illustration of label shift with a toy univariate distribution is shown in Fig 1. Below, we show how to leverage the label shift assumption for changepoint detection.

### Sequential changepoint detection

To detect a change in the unlabeled sequence *X*_1_, *X*_2_, …, classical changepoint detection procedures use a recursive detection statistic

$$\begin{array}{c} R_{t}^{x}=\Psi \left(R_{t-1}\right)\lambda \left(X_{t}\right), \end{array}$$ (3)

where λ(*X*_*t*_) = *f*_0,*X*_(*X*_*t*_)/*f*_∞,*X*_(*X*_*t*_) is the likelihood ratio at time *t*, Ψ is an update function, and the initial value is $R_{0}^{x}=x$. For example, the CUSUM procedure has Ψ(*r*) = max{1, *r*} and *x* = 1, while the Shiryaev-Roberts procedure has Ψ(*r*) = 1 + *r* and *x* = 0 [14]. A change is detected when $R_{t}^{x}$ crosses a pre-specified threshold $A>R_{0}^{x}$, with stopping time

$$\begin{array}{c} T^{x}\left(A\right)=\text{inf}\left\{t\geq 1:R_{t}^{x}\geq A\right\}. \end{array}$$ (4)

However, the pre- and post-change data distributions are rarely known in practice, and a variety of nonparametric alternatives have been proposed. Several authors have adapted nonparametric hypothesis tests to the changepoint detection problem, such as Kolmogorov-Smirnov tests [15], Cramer-von-Mises tests [16], and graph-based nearest-neighbors tests [17, 18]. Others have replaced the likelihood ratio λ with an estimate $\hat{\lambda }$, to detect specific shifts in the mean or variance [19–23] or a change to a stochastically larger/smaller distribution [24–27]. Several papers have also estimated λ using samples from the pre- and post-change distributions, either with nonparametric estimation of the densities [28] or by directly estimating the ratio [29–35].

In general, we expect that samples from the post-change distribution are unavailable until the (unknown) changepoint occurs. In the label shift case, however, the difficulty of having samples from the post-change distribution is reduced to knowing the post-change marginal distribution of *Y* (see Eq (1)). In this manuscript, we propose a simple estimate of the likelihood ratio that leverages the label shift assumption.

### Operating characteristics

The performance of sequential detection procedures, with stopping time *T*^*x*^(*A*) at threshold *A*, is typically assessed by two operating characteristics, the *average time to false alarm* $E_{\infty }\left(T^{x}\left(A\right)\right)$ (also called the *average run length*, or ARL), and the *average detection delay* $E_{0}\left(T^{x}\left(A\right)\right)$, which are expected stopping times under the pre- and post-change distributions respectively. The goal is to minimize the average detection delay, subject to a lower bound on the average time to false alarm, and the CUSUM and Shiryaev-Roberts procedures are known to be optimal or approximately optimal for this problem [36–38]. We therefore compare average detection delay and average time to false alarm as a way to assess procedures in this manuscript.

### Proposed method

Detection procedures which are at least approximately optimal for detecting a change in the unlabeled sequence *X*_1_, *X*_2_, … require the likelihood ratio λ(*X*_*t*_) = *f*_0,*X*_(*X*_*t*_)/*f*_∞,*X*_(*X*_*t*_). While this likelihood ratio is hard to estimate in general, under the label shift assumption the likelihood ratio has a simple expression:

$$\begin{array}{c} \lambda \left(x\right)=\frac{f_{0,X}\left(x\right)}{f_{\infty ,X}\left(x\right)}=(\frac{\pi_{0}}{\pi_{\infty }}-\frac{1-\pi_{0}}{1-\pi_{\infty }})P_{\infty }\left(Y=1|X=x\right)+\frac{1-\pi_{0}}{1-\pi_{\infty }}, \end{array}$$ (5)

with pre- and post-change proportions *π*_∞_ = *P*_∞_(*Y* = 1) and *π*_0_ = *P*_0_(*Y* = 1). Our proposed method for detecting label shift is straightforward: use labeled training data to estimate *P*_∞_(*Y* = 1|*X* = *x*). Since label shift is a concern precisely because we wish to apply a classifier to new data, the existence of labeled training data is no burden.

Let $\left(X_{1}^{\prime },Y_{1}^{\prime }\right),\ldots ,\left(X_{m}^{\prime },Y_{m}^{\prime }\right)\overset{iid}{∼}P_{\infty }$ denote our labeled training set (from the pre-change distribution), and suppose we use the labeled training set to train a classifier A(·) with $A\left(x\right)={\hat{P}}_{\infty }\left(Y=1|X=x\right)\in \left[0,1\right]$. For example, A could be a logistic regression classifier, a random forest, or a neural network. Given *π*_∞_ and *π*_0_, our estimated likelihood ratio is

$$\begin{array}{c} {\hat{\lambda }}_{A,m}\left(x\right)=(\frac{\pi_{0}}{\pi_{\infty }}-\frac{1-\pi_{0}}{1-\pi_{\infty }})A\left(x\right)+\frac{1-\pi_{0}}{1-\pi_{\infty }}, \end{array}$$ (6)

where the subscripts A and *m* denote dependence on the classifier and the size of the training set.

#### Label shift detection with known *π*_0_

**Fig 2:**
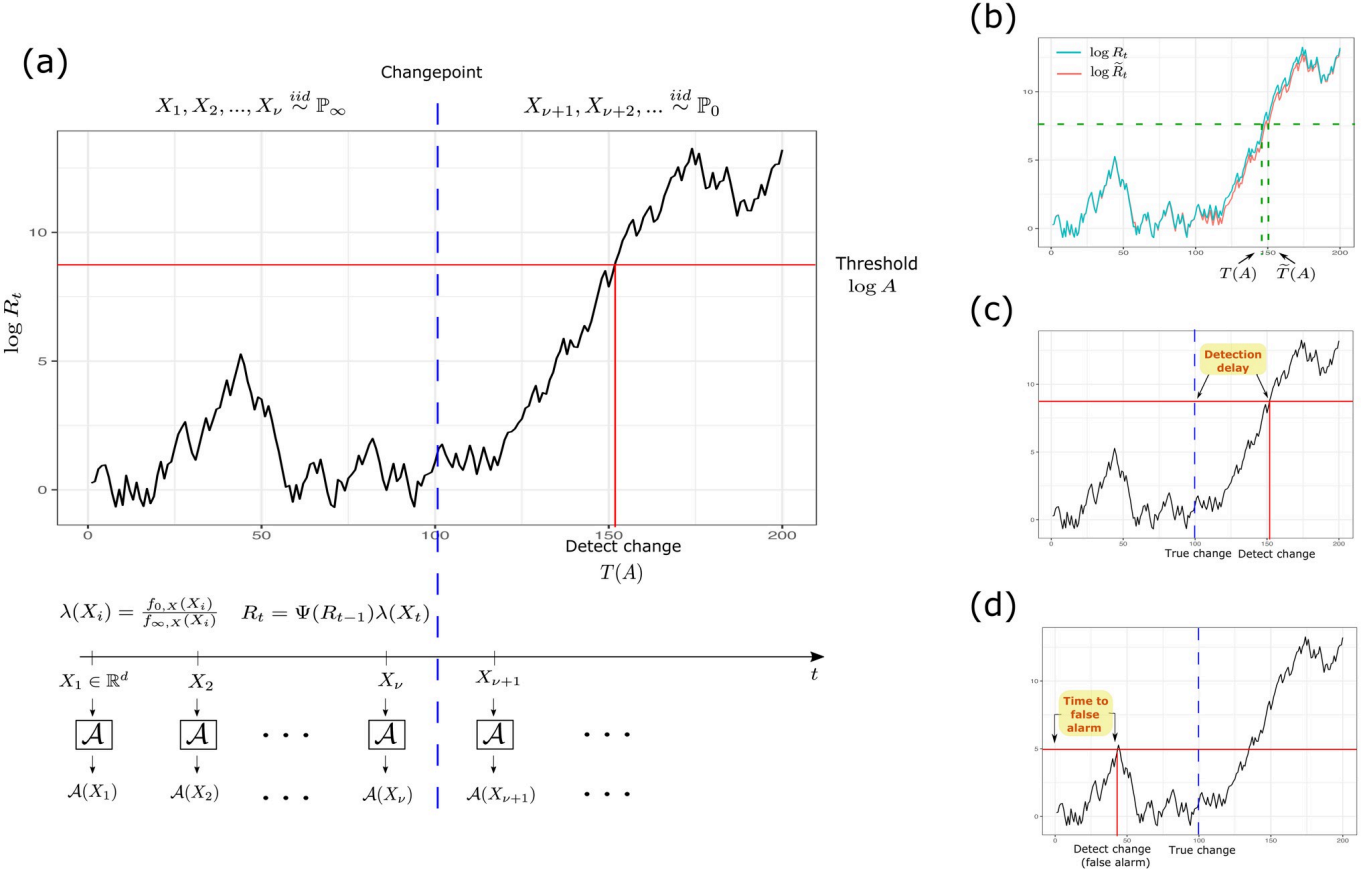
Overview of sequential label shift detection with classifier predictions. **(a)** Data *X*_1_, *X*_2_, … is observed from the pre-change distribution $P_{\infty }$ and the post-change distribution $P_{0}$. At each time *t*, a prediction $A(X_{t})$ is made. If *f*_0,*X*_ and *f*_∞,*X*_ are known, then a detection statistic *R*_*t*_ can be calculated using the likelihood ratio λ(*X*_*t*_). A change is detected when *R*_*t*_ ≥ *A* (or equivalently log *R*_*t*_ ≥ log *A*). **(b)** When the true likelihood ratio λ is unknown, we can use an estimate $\hat{\lambda }$ instead; ${\tilde{R}}_{t}=\Psi \left({\tilde{R}}_{t-1}\right)\hat{\lambda }\left(X_{t}\right)$ is the resulting detection statistic. When $\hat{\lambda }$ is close to λ, the stopping times $\tilde{T}\left(A\right)$ and *T*(*A*) are also expected to be close. **(c)** When a change is detected after the true changepoint *ν*, then *T*(*A*) − *ν* is the detection delay. **(d)** When *T*(*A*) < *ν*, then we have a false alarm, and *T*(*A*) is the time to false alarm.

To detect a label shift change from the unlabeled sequence *X*_1_, *X*_2_, …, we calculate the classifier prediction $A(X_{i})$ for each new observation. Let

$$\begin{array}{c} {\tilde{R}}_{t}^{x}=\Psi \left({\tilde{R}}_{t-1}^{x}\right){\hat{\lambda }}_{A,m}\left(X_{t}\right), \end{array}$$ (7)

and

$$\begin{array}{c} {\tilde{T}}^{x}\left(A\right)=\text{inf}\left\{t\geq 1:{\tilde{R}}_{t}^{x}\geq A\right\}, \end{array}$$ (8)

for some detection threshold *A*. Our goal is to minimize the detection delay $E_{0}\left({\tilde{T}}^{x}\left(A\right)\right)$, while controlling the time to false alarm $E_{\infty }\left({\tilde{T}}^{x}\left(A\right)\right)$. The process is summarized in Fig 2.

Using the estimated likelihood ratio ${\hat{\lambda }}_{A,m}$ will not improve detection performance over the true likelihood ratio λ. However, as classifier performance improves—that is, as $A\left(x\right)\to P_{\infty }\left(Y=1|X=x\right)$—we expect that performance of our detection method will approach the optimal performance with the true likelihood ratio.

**Remark 1**. Using classifier predictions to estimate the likelihood ratio is natural in the label shift setting, as a classifier is already constructed and being applied to make predictions for new data. However, an advantage of the label shift setting is that it supports a variety of other approaches to likelihood ratio estimation. For example, kernel mean matching [34] and uLSIF [30] rely on both pre- and post-change data; under the label shift assumption, a post-change sample can be generated by re-sampling or re-weighting the training data $\left(X_{1}^{\prime },Y_{1}^{\prime }\right),\ldots ,\left(X_{m}^{\prime },Y_{m}^{\prime }\right)$ when *π*_0_ is known. We compare this approach to our classifier-based likelihood ratio estimate in simulations below.

#### Label shift detection with unknown *π*_0_

The estimated likelihood ratio in Eq (6) requires the post-change fraction of positive cases *π*_0_. While we have access to labeled pre-change training data $\left(X_{1}^{\prime },Y_{1}^{\prime }\right),\ldots ,\left(X_{m}^{\prime },Y_{m}^{\prime }\right)$ to train our classifier A, we do not expect a sample of post-change data (labeled or not), and so the post-change parameter *π*_0_ may be unknown. To overcome an unknown *π*_0_, we mix over a set Π_0_ ⊂ [0, 1] of potential values for the post-change parameter, with a weight distribution *w*. Here we are inspired by the work of [39], which deals with the computational complexity involved in the integration by considering a window-limited approach that uses only a fixed number of the most recent observations. Let Π_0_ be the set of possible values for *π*_0_, and let *w*(*π*_0_) be a density on Π_0_. Each potential *π*_0_ results in a different likelihood ratio function $\lambda_{\pi_{0}}$. Lai defines a CUSUM-type mixture stopping rule with detection statistic *R*_*t*,*w*_ and stopping time *T*_*w*_(*A*) [39]:

$$\begin{array}{c} R_{t,w}=\underset{t-m_{\alpha }\leq k\leq t}{\text{max}}\int_{\Pi_{0}}\prod_{i=k}^{t}\lambda_{\pi_{0}}\left(X_{i}\right)w\left(\pi_{0}\right)d\pi_{0}\;T_{w}\left(A\right)=\text{inf}\left\{t\geq 1:R_{t,w}\geq A\right\}, \end{array}$$ (9)

where *m*_*α*_ is the window size. In our label shift setting, we have

$$\begin{array}{c} \lambda_{\pi_{0}}\left(x\right)=\frac{\pi_{0}f_{\infty ,X|Y=1}\left(x\right)+\left(1-\pi_{0}\right)f_{\infty ,X|Y=0}\left(x\right)}{\pi_{\infty }f_{\infty ,X|Y=1}\left(x\right)+\left(1-\pi_{\infty }\right)f_{\infty ,X|Y=0}\left(x\right)}. \end{array}$$ (10)

For each *π*_0_, we replace $\lambda_{\pi_{0}}$ with its estimate ${\hat{\lambda }}_{\pi_{0},A,m}$ from Eq (6), yielding the detection statistic ${\tilde{R}}_{t,w}$ and stopping time ${\tilde{T}}_{w}\left(A\right)$:

$$\begin{array}{c} {\tilde{R}}_{t,w}=\underset{t-m_{\alpha }\leq k\leq t}{\text{max}}\int_{\Pi_{0}}\prod_{i=k}^{t}{\hat{\lambda }}_{\pi_{0},A,m}\left(X_{i}\right)w\left(\pi_{0}\right)d\pi_{0}\;{\tilde{T}}_{w}\left(A\right)=\text{inf}\left\{t\geq 1:{\tilde{R}}_{t,w}\geq A\right\}. \end{array}$$ (11)

When $A(X_{i})$ is a good estimate of $P_{\infty }\left(Y_{i}=1|X_{i}\right)$, we expect ${\tilde{T}}_{w}\left(A\right)$ (11) to be close to *T*_*w*_(*A*) (9).

**Remark 2**. An alternative to mixing over Π_0_ is to maximize over possible values of *π*_0_ at each time step. This is the generalized likelihood ratio (GLR) approach, and has also been studied in previous research (see, e.g., [40]). For exponential families, some optimality properties of the GLR have been shown, but it is typically harder to control the average run length to false alarm [41]. Another option is to perform detection with a worst-case $\pi_{0}^{*}\in \Pi_{0}$ [42], which provides a worst-case bound on detection delay.

### Simulations

We investigate the empirical performance of the classifier-based label shift detection procedure with the likelihood ratio estimate in Eq (6). Our likelihood ratio estimate depends on a classifier, and for simplicity we will use an LDA classifier, since it is easy to control whether the LDA assumptions are satisfied. For comparison, we consider several other detection procedures, which represent different approaches to changepoint detection. These procedures are summarized below and in Table 1. Because our label shift detection procedure is designed specifically for label shift, it leverages more information than the other nonparametric detection procedures. In particular, as summarized in Table 1, estimating the likelihood ratio with Eq (6) assumes that the label shift assumption holds, and the classifier A(·) performs well. Through simulations, we show that detection with Eq (6) outperforms the other nonparametric procedures when these assumptions are met, and can still perform well when the assumptions are violated. While we use a simple setting for simulations, we also apply the same methods to detect a change in dengue prevalence using the data and classifier from [3], with similar results to our simulations in this section.

**Table 1:** Comparison of the information used by each changepoint detection procedure considered in simulations.

Information leveraged	Classifier CUSUM	Optimal CUSUM	uLSIF CUSUM	CPM (classifier)	CPM (divergence)	kNN
True labels		✓				
Label shift	✓		✓			
Good classifier	✓			✓		
Training data	✓		✓	✓	✓	✓

CPM and kNN are more general than the classifier CUSUM procedure, but as a result they leverage less information. If the label shift assumption holds and the classifier performs well, we expect the classifier-based CUSUM method to outperform these more general procedures.

We compare the following methods:

**Classifier-based CUSUM** This is the nonparametric method proposed in the Methods, with likelihood ratio estimate (6). For the purposes of simulations, A in Eq (6) is an LDA classifier. Here we use a CUSUM procedure, so Ψ(*r*) = max{1, *r*}.
**Optimal CUSUM** The optimal CUSUM procedure [43] uses the true likelihood ratio, and can be implemented when the true likelihood ratio is known.
**uLSIF CUSUM** uLSIF [30] is a nonparametric method for estimating the likelihood ratio, by maximizing an empirical divergence. As described above, uLSIF can be used with training data under the label shift assumption by re-weighting or re-sampling training points, but it does not exploit the label shift structure of the likelihood ratio. A variety of similar density ratio estimation approaches exist, including KLIEP and kernel mean matching [32, 34, 44], and we take uLSIF as a representative. Here we use the densratio package [45] to implement uLSIF, and employ the resulting estimate in a CUSUM procedure.
**CPM** [12] perform nonparametric label shift detection using the CPM framework described in [46, 47]. The CPM framework detects changes in a sequence of univariate data using repeated nonparametric tests; [12] applied repeated Cramer–von-Mises tests to a sequence of cosine divergences calculated between new data and training data. We evaluate CPM applied to both the **classifier** predictions and the cosine **divergences** used by [12]. CPM stopping times are calculated with the cpm package [47].
**kNN** [17, 18] propose a sequential graph-based *k*-nearest neighbors (kNN) detection procedure, based on repeated nearest-neighbor two-sample tests in a sliding window. Note that while the kNN approach uses training data, only a fixed window of data is considered. Similar to some parameters in [18], we set the window size to 200 and the number of nearest neighbors to *k* = 5. Stopping times are calculated with the gStream package [48].

#### Metrics

Performance of each detection procedure is measured by detection delay, calculated as $E_{0}\left[T\right]$ (for CUSUM procedures, this corresponds to Lorden’s [36] detection delay). As is standard, we compare detection delays with each method calibrated to have the same average run length $E_{\infty }\left[T\right]$. Here we use $E_{\infty }\left[T\right]=500$, which is a common value in the sequential detection literature. Expected stopping times are estimated via Monte Carlo simulation.

#### Scenarios

Under the label shift assumption, the classifier-based CUSUM procedure uses classifier predictions $A(X_{i})$ to estimate the likelihood ratio. To compare performance of the different detection procedures, we use two different simulation scenarios. In the first scenario, we change the training sample size and the performance of the classifier (by changing the distribution of the data *X*_*i*_ and violating LDA assumptions). In the second scenario, we change the performance of the classifier and the suitability of the label shift assumption.

**Scenario 1**: Data is generated as *X*|(*Y* = 0) ∼ *N*(***μ***_**0**_, **Σ**_**0**_) and *X*|(*Y* = 1) ∼ *N*(***μ***_**1**_, **Σ**_**1**_). In all simulations, *π*_∞_ = 0.4, *π*_0_ = 0.7, ***μ***_**0**_ = [0, 0], ***μ***_**1**_ = [1.5, 1.5], and **Σ**_**0**_ = ***I***. Training data $\left(X_{1}^{\prime },Y_{1}^{\prime }\right),\ldots ,\left(X_{m}^{\prime },Y_{m}^{\prime }\right)$ is simulated from the pre-change distribution, and used to train the LDA classifier, estimate the uLSIF likelihood ratio, and startup the CPM and kNN detection statistics. We consider *m* ∈ {200, 1000, 5000}, and $\Sigma_{1}\in \{I,[\begin{array}{cc} 2 & 0.1\\ 0.1 & 2 \end{array}],[\begin{array}{cc} 4 & 0.5\\ 0.5 & 4 \end{array}]\}$.
**Scenario 2**: Pre-change data is generated as *X*|(*Y* = 0) ∼ *N*(***μ***_**∞**,**0**_, **Σ**_**0**_) and *X*|(*Y* = 1) ∼ *N*(***μ***_**∞**,**1**_, **Σ**_**1**_), while post-change data is generated as *X*|(*Y* = 0) ∼ *N*(***μ***_**0**,**0**_, **Σ**_**0**_) and *X*|(*Y* = 1) ∼ *N*(***μ***_**0**,**1**_, **Σ**_**1**_). In all simulations, *π*_∞_ = 0.4, *π*_0_ = 0.7, ***μ***_**∞**,**0**_ = [0, 0], ***μ***_**∞**,**1**_ = [1.5, 1.5], and **Σ**_**0**_ = ***I***. Training data $\left(X_{1}^{\prime },Y_{1}^{\prime }\right),\ldots ,\left(X_{1000}^{\prime },Y_{1000}^{\prime }\right)$ is simulated from the pre-change distribution, and used to train the LDA classifier, estimate the uLSIF likelihood ratio, and startup the CPM and kNN detection statistics. We consider $\Sigma_{1}\in \{I,[\begin{array}{cc} 2 & 0.1\\ 0.1 & 2 \end{array}],[\begin{array}{cc} 4 & 0.5\\ 0.5 & 4 \end{array}]\}$ and the following pairs for ***μ***_**0**,**0**_ and ***μ***_**0**,**1**_: ***μ***_**0**,**0**_ = [0.5, 0.5] and ***μ***_**0**,**1**_ = [1, 1]; ***μ***_**0**,**0**_ = [0.75, 0.75] and ***μ***_**0**,**1**_ = [0.75, 0.75]; and ***μ***_**0**,**0**_ = [1, 1] and ***μ***_**0**,**1**_ = [0.5, 0.5].

### Case study: Dengue fever

#### Data

Data comes from [3], who collected information on 5720 febrile patients aged 15 or younger in three Vietnamese hospitals. Of these patients, 30% had dengue. The authors recorded their true dengue status (using a gold-standard test), the results of an NS1 rapid antigen test, and a variety of physical measurements for classification with a logistic regression classifier. This dataset is anonymized and publicly available, and neither author of the present study was involved in [3], nor did we have any means of identifying any of the patients in these studies.

#### Classifier

We use 1000 patients as training data for the classifier, and save the rest for evaluating our classifier and estimating changepoint detection performance. With the training set, we construct a logistic GAM classifier to predict true dengue status with the following covariates: vomiting (yes/no), skin bleeding (yes/no), BMI, age, temperature, white blood cell count, hematocrit, and platelet count. As in [3], the ROC curve has an AUC of approximately 0.8. The explanatory variables chosen here were included because they fit the label shift assumption in exploratory data analysis, and previous research [3, 49] demonstrates that adding additional variables to the model does not improve predictive performance or generalizability to new populations. The use of logistic regression (and variants) is also common in the dengue prediction literature [3, 4, 49–51], found that logistic regression methods were comparable or outperformed other approaches.

#### Scenarios

To assess change detection, we simulate a change in the prevalence of dengue by resampling the 4720 patients not used for training. As the group of patients in the study aims to represent the population of patients who would be tested for dengue, we take the sample proportion of 30% as our baseline dengue prevalence among patients who would be tested. The degree of change in this prevalence, when an outbreak occurs, depends on the magnitude of the outbreak and the baseline prevalence in the population. Magnitude of change varies; for example, Hanoi, Vietnam saw roughly a five-fold increase in 2009 and 2015 [52, 53], while Kaohsiung City, Taiwan saw a 15-fold increase in 2014 [7]. Baseline prevalence in the full population varies depending on location—for example [5], shows approximately 1 in 1 million for certain areas of Thailand, whereas [7] show roughly 1 in 10000 on average in Taiwan. For Vietnam [52], report roughly 1 in 10000 to 1 in 1000 in Hanoi, with a peak of 384 per 100000 in 2009. For our purposes, we consider two label shift changes in prevalence:

**Abrupt change**: We simulate an abrupt 5-fold increase, and take the baseline prevalence in the population to be roughly 1 in 10000. Applying Bayes rule, this gives a post-change prevalence of about 68% in our study population, and so we simulate a change from 30% to 68% and assess our ability to detect this shift.
**Gradual change**: When the change occurs, prevalence increases gradually, rather than abruptly. Here, prevalence in the study population changes smoothly from 30% to 68% over the course of 100 observations.

#### Methods for comparison in dengue setting

We compare the methods discussed above in the simulations to detect the change in dengue prevalence. The classifier CUSUM detection procedure is implemented using Eq (6) with A(X) the predicted probabilities from the dengue classifier described above. We also compare CUSUM with binarized predictions, using both a threshold of 0.5 and the threshold, 0.33, which maximizes sensitivity + specificity. The optimal CUSUM procedure uses the true dengue status, which is observable if gold-standard tests are available, and we also include CUSUM with binary predictions from the NS1 rapid antigen tests, which again may not be available. The rapid test has a specificity of approximately 99% and a sensitivity of 70% [3], compared with a specificity and sensitivity of 82% and 70% for the binarized classifier at threshold 0.33. As in the simulations, we also compare CPM using the classifier predicted probabilities, and CPM with divergences. uLSIF was considered but failed to consistently estimate the likelihood ratio, while kNN was not considered because it performed worse than the other methods examined. Finally, as the post-change parameter is typically unknown, we include the mixing procedure described in Eq (11). We use Π_0_ = [0.6, 0.8], which corresponds to a 3.5-fold to 9-fold increase in prevalence.

For the abrupt change scenario, all methods are compared. For the gradual change, we compare the mixture CUSUM procedure to CPM with classifier predictions, as these two methods perform well at detecting an abrupt change and do not require knowledge of the post-change parameter, and we include optimal CUSUM for reference.

## Results

### Simulation results

Table 2 shows the results for Scenario 1, when the label shift assumption holds. We can see that when the LDA assumptions are met (specifically **Σ**_**1**_ = **Σ**_**0**_ = ***I***), LDA performs very close to the optimal CUSUM procedure, as we would predict. Performance of the LDA detection procedure relative to the optimal CUSUM procedure declines as the assumption that **Σ**_**1**_ = **Σ**_**0**_ is violated, but is still better than the other nonparametric methods. This suggests that if the label shift assumption holds, the likelihood ratio estimate in Eq (6) is a good choice for detecting the change, even if the classifier is mis-specified. Detection with the uLSIF procedure improves with training sample size *m*, as it becomes easier to estimate the likelihood ratio function and variability in the likelihood ratio estimate decreases. CPM also performs better as the sample size increases, as training data is used to construct the detection statistic. While the kNN method makes no assumptions about the change or the distribution of data, the cost of this flexibility is a decrease in detection performance.

**Table 2:** Simulation results for Scenario 1.

Σ_1_	*m*	Detection delay when $E_{\infty }\left[T\right]\approx 500$					
		Classifier CUSUM	Optimal CUSUM	uLSIF CUSUM	CPM (classifier)	CPM (divergence)	kNN
$\left[\begin{array}{cc} 1 & 0\\ 0 & 1 \end{array}\right]$	200	28.8 (0.59)	29.0 (0.23)	33.8 (5.62)	46.9 (1.45)	51.5 (1.72)	≥ 155 (3.46)
	1000	28.8 (0.26)		33.4 (1.51)	35.0 (0.81)	37.0 (0.85)	
	5000	28.8 (0.10)		31.5 (1.11)	32.6 (0.76)	33.7 (0.81)	
$\left[\begin{array}{cc} 2 & 0.1\\ 0.1 & 2 \end{array}\right]$	200	34.3 (0.96)	33.1 (0.28)	49.0 (11.4)	61.2 (2.24)	69.5 (2.67)	≥ 168 (3.58)
	1000	34.0 (0.40)		43.3 (2.39)	42.4 (1.07)	46.6 (1.18)	
	5000	34.0 (0.16)		38.8 (2.44)	38.1 (0.94)	41.7 (1.03)	
$\left[\begin{array}{cc} 4 & 0.5\\ 0.5 & 4 \end{array}\right]$	200	41.9 (1.44)	33.4 (0.29)	164 (47.3)	74.6 (2.82)	94.8 (3.56)	≥ 176 (3.59)
	1000	41.8 (0.60)		73.3 (7.05)	52.1 (1.46)	62.4 (1.74)	
	5000	41.9 (0.25)		54.2 (5.19)	51.2 (1.36)	58.5 (1.55)	

Performance of each procedure is measured by detection delay, calculated as $E_{0}\left[T\right]$. The estimated detection delay from Monte Carlo simulation is reported, with the standard error in parentheses. For the kNN procedure, a window of size 200 is used, so only 200 training points are considered. In the case of kNN, if a change is not detected within the sliding window, windows after time point 200 will consist of only post-change observations, so for computational purposes a fixed number of post-change observations is simulated and we report a lower bound on the detection delay.

Table 3 shows the results for Scenario 2, when the label shift assumption is violated. When the label shift assumption is approximately true (***μ***_**0**,**0**_ = [0.5, 0.5] and ***μ***_**0**,**1**_ = [1, 1]), we can see that LDA detection is comparable to uLSIF and CPM. However, the LDA procedure is more sensitive to large departures from the label shift assumption, for which methods with fewer assumptions perform better. Overall, CPM with classifier predictions performs well, as the classifier predictions are a useful summary of the data even when label shift doesn’t hold.

**Table 3:** Simulation results for Scenario 2.

Σ_1_	Post-change distribution	Detection delay when $E_{\infty }\left[T\right]\approx 500$					
		Classifier CUSUM	Optimal CUSUM	uLSIF CUSUM	CPM (classifier)	CPM (divergence)	kNN
$\left[\begin{array}{cc} 1 & 0\\ 0 & 1 \end{array}\right]$	$\;\begin{array}{cc} \mu_{0,0} & =[0.5,0.5]\\ \mu_{0,1} & =[1,1] \end{array}$	70.2 (1.68)	24.1 (0.17)	55.8 (4.49)	64.4 (1.27)	66.4 (1.40)	≥ 130 (3.22)
	$\;\begin{array}{cc} \mu_{0,0} & =[0.75,0.75]\\ \mu_{0,1} & =[0.75,0.75] \end{array}$	184 (7.49)	22.9 (0.16)	117 (14.6)	92.1 (1.64)	96.0 (1.78)	≥ 131 (3.30)
	$\;\begin{array}{cc} \mu_{0,0} & =[1,1]\\ \mu_{0,1} & =[0.5,0.5] \end{array}$	541 (17.4)	28.9 (0.21)	282 (3.23)	177 (3.18)	193 (3.55)	≥ 161 (3.57)
$\left[\begin{array}{cc} 2 & 0.1\\ 0.1 & 2 \end{array}\right]$	$\;\begin{array}{cc} \mu_{0,0} & =[0.5,0.5]\\ \mu_{0,1} & =[1,1] \end{array}$	70.9 (1.66)	34.3 (0.27)	67.9 (6.17)	74.6 (1.78)	79.4 (1.92)	≥ 173 (3.60)
	$\;\begin{array}{cc} \mu_{0,0} & =[0.75,0.75]\\ \mu_{0,1} & =[0.75,0.75] \end{array}$	123 (3.82)	31.4 (0.26)	101 (11.5)	109 (2.69)	120 (3.03)	≥ 177 (3.59)
	$\;\begin{array}{cc} \mu_{0,0} & =[1,1]\\ \mu_{0,1} & =[0.5,0.5] \end{array}$	225 (8.56)	30.0 (0.24)	166 (20.4)	196 (5.42)	217 (6.15)	≥ 177 (3.60)
$\left[\begin{array}{cc} 4 & 0.5\\ 0.5 & 4 \end{array}\right]$	$\;\begin{array}{cc} \mu_{0,0} & =[0.5,0.5]\\ \mu_{0,1} & =[1,1] \end{array}$	73.8 (1.67)	29.8 (0.24)	87.6 (10.1)	77.0 (1.98)	88.7 (2.49)	≥ 170 (3.57)
	$\;\begin{array}{cc} \mu_{0,0} & =[0.75,0.75]\\ \mu_{0,1} & =[0.75,0.75] \end{array}$	103 (2.78)	22.9 (0.19)	101 (11.9)	88.9 (2.55)	101 (3.08)	≥ 152 (3.50)
	$\;\begin{array}{cc} \mu_{0,0} & =[1,1]\\ \mu_{0,1} & =[0.5,0.5] \end{array}$	146 (4.58)	18.1 (0.15)	120 (15.3)	107 (3.12)	122 (3.75)	≥ 120 (3.20)

Performance of each procedure is measured by detection delay, calculated as $E_{0}\left[T\right]$. The estimated detection delay from Monte Carlo simulation is reported, with the standard error in parentheses. For the kNN procedure, a window of size 200 is used, so only 200 training points are considered. In the case of kNN, if a change is not detected within the sliding window, windows after time point 200 will consist of only post-change observations, so for computational purposes a fixed number of post-change observations is simulated and we report a lower bound on the detection delay.

### Case study results: Detecting a dengue outbreak

#### Abrupt change

**Fig 3:**
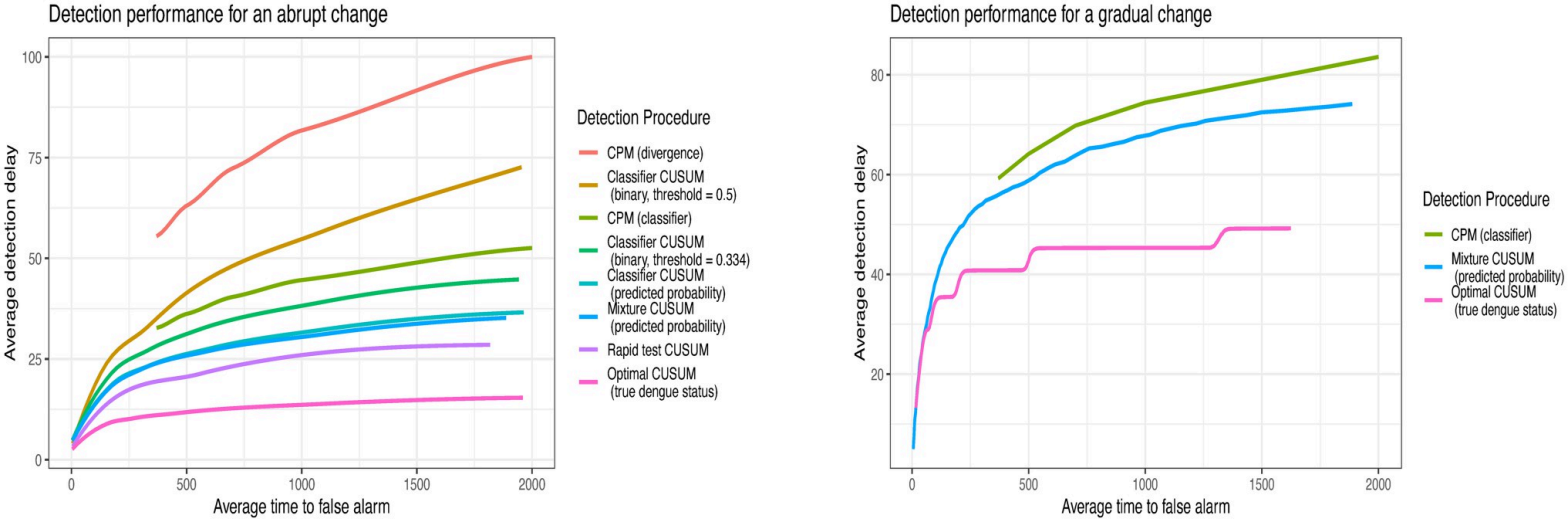
Performance of changepoint detection procedures for a simulated change in dengue prevalence. Left: Comparison of detection performance for CUSUM procedures using different detection procedures, for a change in dengue prevalence from *π*_∞_ = 0.3 to *π*_0_ = 0.68. For ease, the method labels for the plot are displayed in descending order of detection delay. Right: Comparison of detection performance when *π*_0_ changes gradually from 0.3 to 0.68.

Fig 3 and Table 4 show the relationship between $E_{\infty }\left[T\right]$ (average time to false alarm) and $E_{0}\left[T\right]$ (average detection delay) for each method (uLSIF is not shown in Fig 3 because the detection delays are too large). As expected, the true dengue status and the rapid antigen test give the best detection performance. The predicted probabilities outperform the binarized predictions, as binarization throws away information on the likelihood ratio. The two binarized predictions are close, but the optimal threshold—which maximizes sensitivity + specificity − performs better. Mixture CUSUM and CUSUM with the predicted probabilities perform equally well, likely because all *π*_0_ ∈ Π_0_ = [0.6, 0.8] provide similar results. While CPM performs worse than CUSUM with predicted probabilities, it still provides a competitive alternative that requires no assumptions on the post-change prevalence. uLSIF has difficulty estimating the likelihood ratio, and performs substantially worse than the other methods.

**Table 4:** Comparison of method performance for detecting an abrupt change in dengue prevalence.

Method	Detection delay for three values of $E_{\infty }\left[T\right]$		
	$E_{\infty }\left[T\right]=500$	$E_{\infty }\left[T\right]=700$	$E_{\infty }\left[T\right]=1000$
Optimal CUSUM	11.73 (0.06)	12.56 (0.06)	13.62 (0.07)
Rapid test CUSUM	19.46 (0.15)	23.21 (0.20)	24.66 (0.20)
Mixture CUSUM	25.56 (0.68)	27.52 (0.70)	30.04 (0.75)
Classifier CUSUM (predicted probability)	26.28 (0.16)	29.06 (0.17)	31.67 (0.18)
Classifier CUSM (binary, threshold = 0.33)	30.54 (0.22)	33.58 (0.23)	37.54 (0.26)
CPM (classifier)	36.2 (0.28)	40.4 (0.30)	44.6 (0.32)
Classifier CUSUM (binary, threshold = 0.5)	41.22 (0.37)	49.72 (0.45)	56.04 (0.51)
CPM (divergence)	63.0 (0.54)	72.3 (0.60)	81.7 (0.67)
uLSIF CUSUM	1295 (88)	1745 (111)	2305 (141)

Performance of each procedure is assessed by average detection delay ($E_{0}\left[T\right]$), calculated at three different values of average run length ($E_{\infty }\left[T\right]$). The estimated detection delay from Monte Carlo simulation is reported, with the standard error in parentheses.

#### Gradual change

Fig 3 shows the relationship between $E_{\infty }\left[T\right]$ and $E_{0}\left[T\right]$ for each method. Detection delays are longer for all methods under gradual change than abrupt change, because the magnitude of change is initially smaller. However, each method can raise an alarm reasonably quickly. This is valuable because real changes in prevalence are expected to be continuous, rather than an abrupt switch from one prevalence to another. While the classic CUSUM procedure, and the nonparametric methods discussed in this paper, are designed to detect an abrupt change, Fig 3 demonstrates that these methods are sensitive to other changes too.

## Discussion

Many previous papers have proposed classifiers to help diagnose dengue fever. When these classifiers are applied sequentially over time, it is important to detect any change in the distribution of the data. First, distributional shifts can affect the validity of classifier predictions, and second, a change in distribution may suggest a problem like a disease outbreak. In this paper, we consider procedures for detecting label shift, which can occur when the prevalence of a disease changes over time, but the symptoms of the disease remain the same.

As we focus on detecting changes in classification data, it is natural to use the classifier predictions in our detection procedure. Here we propose a simple, nonparametric sequential changepoint detection method that uses the classifier predictions to approximate the true likelihood ratio (6). Our procedure requires no additional estimation or training, assuming only that a reasonable value of the post-change prevalence *π*_0_ can be specified. Furthermore, when this post-change parameter is unknown, we combine our nonparametric procedure with Lai’s mixture CUSUM approach [39], and mix over the unknown prevalence.

Performance of the detection procedure then depends directly on classifier performance. Through simulations, we illustrate that our proposed detection procedure outperforms other nonparametric methods when the label shift assumption holds, and still achieves comparable performance when the the label shift assumption is violated. The same holds true when these methods are applied to real dengue classification data, in which we apply the classifier described in [3] to detect a simulated dengue outbreak. First, we see that improved classifier performance results in improved detection performance—if the gold standard dengue test is unavailable, only the NS1 rapid antigen test (which has better specificity than the classifier from [3]) outperforms our proposed procedure. Second, other nonparametric procedures respond more slowly to the outbreak, because they leverage less information about a change in prevalence.

Due to limitations of the available data, the changes in dengue prevalence in this manuscript have been simulated to illustrate the proposed changepoint detection procedure. A valuable direction for future research would be to monitor features of dengue diagnosis over the course of population outbreaks and assess the power of the label shift assumption used here. However, while the label shift assumption might not hold exactly in real data [9], found that testing for label shift is still a useful way for finding more general changes in distribution. This is supported by our simulation results, in which our label shift detection procedure still performs well under mild violations of the label shift assumption.

We also note that the classifier considered in the motivating example in this manuscript was trained on data from [3], which is specific to several hospitals in Vietnam. To assess the generalizability of dengue classifiers between different populations [49], studied classifier performance across five different publicly available dengue datasets, and their results show that dengue predictions do not always generalize to new populations. Given the importance of classifier performance in the changepoint detection procedure presented here, we recommend that for now, classifiers be constructed on relevant training data from the population of interest.

Finally, we note that the classic changepoint detection methods discussed here assume a sequence of independent observations. The purpose of this paper is to show how classifier predictions can be incorporated into a changepoint detection procedure based on the likelihood ratio, and classifier predictions could also be used in other detection procedures which rely on the likelihood ratio. Independence may also be a reasonable approximation in many settings. For example, dengue fever is typically transmitted through mosquitoes after a period of about a week, and is not spread by close contact, respiratory droplets, or bodily fluids (unless blood is involved); there may therefore be much less dependence between cases.

## Supporting information

S1 Data Full data and code for all results in this manuscript. Full code for the data analysis and simulations presented in this paper is available at https://github.com/ciaran-evans/label-shift-detection. The data used in the dengue case study was made publicly available by [3], and a copy is provided in the repository with the code.
(ZIP)
