# Supplementary material for: Sequential label shift detection in classification data: An application to dengue fever
Source: PLoS One. 2024 Sep 16;19(9):e0310194. doi: 10.1371/journal.pone.0310194 (PMC11404796; doi:10.1371/journal.pone.0310194)
Supplement: S1 Data — Full code for the data analysis and simulations presented in this paper is available at https://github.com/ciaran-evans/label-shift-detection. The data used in the dengue case study was made publicly available by [3], and a copy is provided in the repository with the code. (ZIP) [file pone.0310194.s001.zip › label-shift-detection/dengue_detection_plots.pdf]

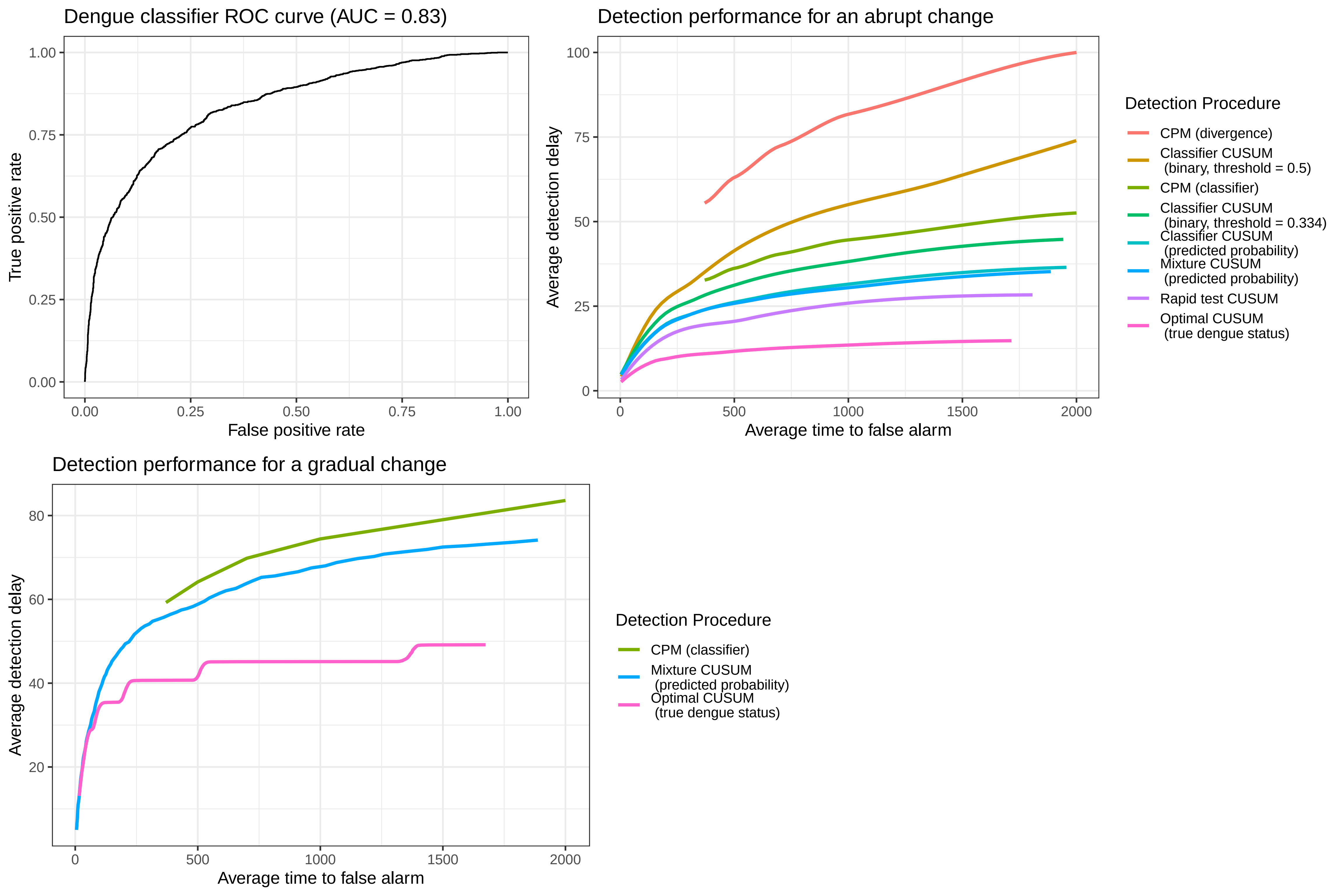

Dengue classifier ROC curve (AUC = 0.83)
True positive rate
False positive rate
0.00
0.25
0.50
0.75
1.00
Detection performance for an abrupt change
Average detection delay
Average time to false alarm
0
25
50
75
100
500
1000
1500
2000
Detection Procedure
CPM (divergence)
Classifier CUSUM (binary, threshold = 0.5)
CPM (classifier)
Classifier CUSUM (binary, threshold = 0.334)
Classifier CUSUM (predicted probability)
Mixture CUSUM (predicted probability)
Rapid test CUSUM
Optimal CUSUM (true dengue status)
Detection performance for a gradual change
Average detection delay
Average time to false alarm
20
40
60
80
Detection Procedure
CPM (classifier)
Mixture CUSUM (predicted probability)
Optimal CUSUM (true dengue status)
