# Supplementary figures and images for: Sequential label shift detection in classification data: An application to dengue fever
Source: PLoS One. 2024 Sep 16;19(9):e0310194. doi: 10.1371/journal.pone.0310194 (PMC11404796; doi:10.1371/journal.pone.0310194)

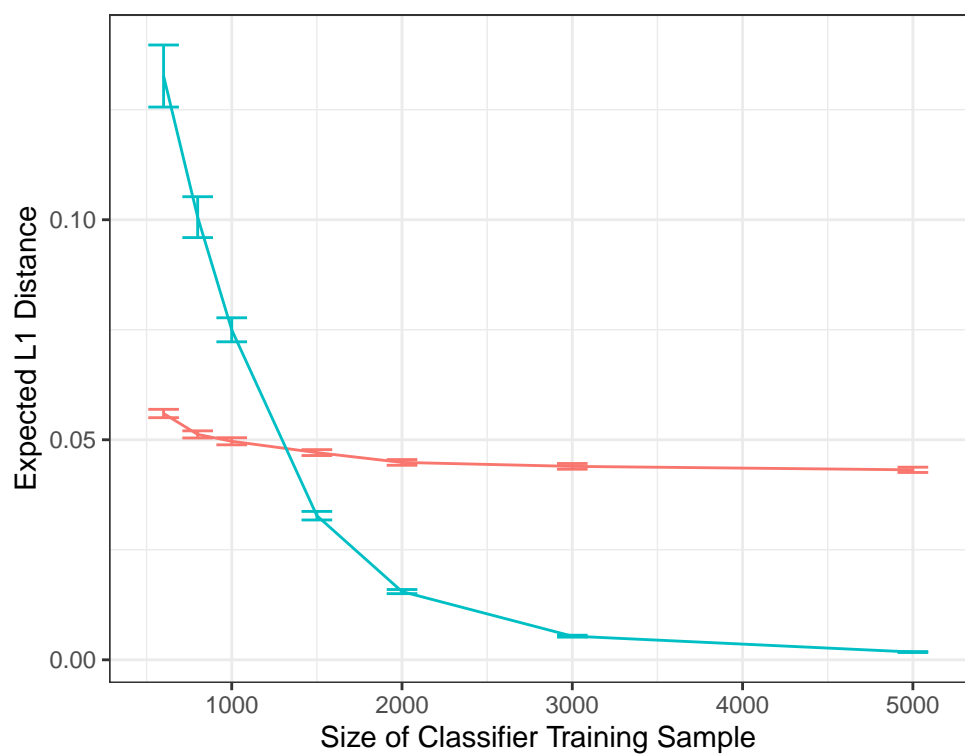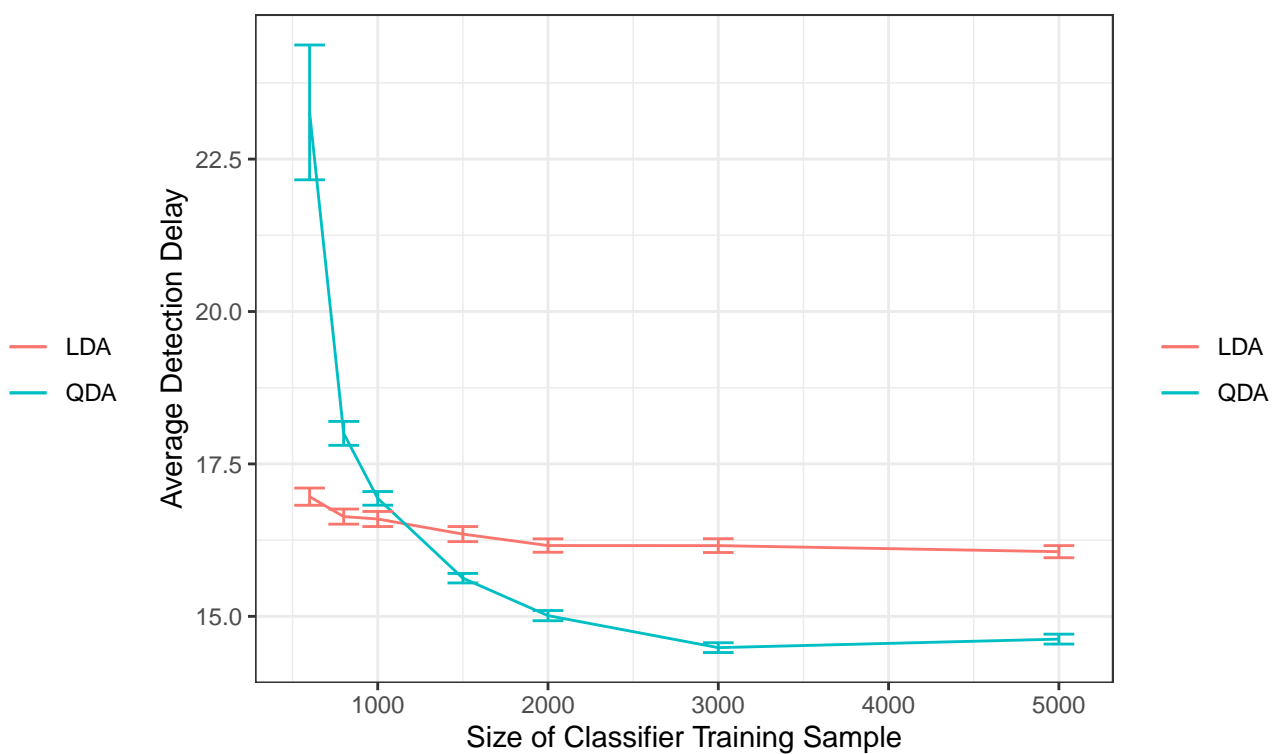

Supplement: S1 Data — Full code for the data analysis and simulations presented in this paper is available at https://github.com/ciaran-evans/label-shift-detection. The data used in the dengue case study was made publicly available by [3], and a copy is provided in the repository with the code. (ZIP) [file pone.0310194.s001.zip › label-shift-detection/lda_qda_classifier_comparison_plot.pdf]
